# Single-Cell DNA Sequencing and Immunophenotypic Profiling to Track Clonal Evolution in an Acute Myeloid Leukemia Patient

**DOI:** 10.3390/biomedicines12010066

**Published:** 2023-12-27

**Authors:** María García-Álvarez, Ana Yeguas, Cristina Jiménez, Alejandro Medina-Herrera, Verónica González-Calle, Montserrat Hernández-Ruano, Rebeca Maldonado, Irene Aires, Cristina Casquero, Inmaculada Sánchez-Villares, Ana Balanzategui, María Eugenia Sarasquete, Miguel Alcoceba, María Belén Vidriales, Marcos González-Díaz, Ramón García-Sanz, María Carmen Chillón

**Affiliations:** 1 Hematology Department, University Hospital of Salamanca (HUS/IBSAL), CIBERONC and Cancer Research Institute of Salamanca-IBMCC (USAL-CSIC), 37007 Salamanca, Spain; mgarca1991@gmail.com (M.G.-Á.); jscris@usal.es (C.J.); amedinah@saludcastillayleon.es (A.M.-H.); vgcalle@saludcastillayleon.es (V.G.-C.); mhernandezru@saludcastillayleon.es (M.H.-R.); rebecamaldonadosanchez@gmail.com (R.M.); iaires.ibsal@saludcastillayleon.es (I.A.); ccasquero.ibsal@saludcastillayleon.es (C.C.); isanchezvillares@saludcastillayleon.es (I.S.-V.); abal@usal.es (A.B.); mealonsos@saludcastillayleon.es (M.E.S.); alcocebasanchez@saludcastillayleon.es (M.A.); mbvidriales@saludcastillayleon.es (M.B.V.); margondi@usal.es (M.G.-D.); mcchillon@saludcastillayleon.es (M.C.C.); 2 Hematology Department, Complejo Asistencial Universitario de Palencia, 34005 Palencia, Spain; ayeguas@saludcastillayleon.es

**Keywords:** acute myeloid leukemia, *FLT3*-ITD, single-cell DNA sequencing, next-generation sequencing, immunophenotype, midostaurin, gilteritinib and clonal evolution

## Abstract

Single-cell DNA sequencing can address the sequence of somatic genetic events during myeloid transformation in relapsed acute myeloid leukemia (AML). We present an *NPM1*-mutated AML patient with an initial low ratio of *FLT3*-ITD (low-risk ELN-2017), treated with midostaurin combined with standard chemotherapy as front-line treatment, and with salvage therapy plus gilteritinib following allogenic stem cell transplantation after relapse. Simultaneous single-cell DNA sequencing and cell-surface immunophenotyping was used in diagnostic and relapse samples to understand the clinical scenario of this patient and to reconstruct the clonal composition of both tumors. Four independent clones were present before treatment: *DNMT3A/DNMT3A/NPM1* (63.9%), *DNMT3A/DNMT3A* (13.9%), *DNMT3A/DNMT3A/NPM1/FLT3* (13.8%), as well as a wild-type clone (8.3%), but only the minor clone with *FLT3*-ITD survived and expanded after therapy, being the most represented one (58.6%) at relapse. *FLT3*-ITD was subclonal and was found only in the myeloid blast population (CD38/CD117/CD123). Our study shows the usefulness of this approach to reveal the clonal architecture of the leukemia and the identification of small subclones at diagnosis and relapse that may explain how the neoplastic cells can escape from the activity of different treatments in a stepwise process that impedes the disease cure despite different stages of complete remission.

## 1. Introduction

Acute myeloid leukemia (AML) is a very aggressive neoplasia with high rates of treatment failure, showing 5-year survival rates that do not exceed 20–40% in older patients [1]. The development of AML is driven by the acquisition of multiple mutations and numerous additional genetic and epigenetic abnormalities by the hematopoietic stem cells and progenitor cells, which disrupt the normal mechanisms of self-renewal, proliferation and differentiation [2,3,4,5]. The gradual incorporation of somatic and germline mutations into disease classification has improved the diagnosis and risk stratification, allowing more precise clinical algorithms [6,7,8]. The recent updated version of the European LeukemiaNet (ELN) recommendations maintains the stratification into three groups but introduces significant changes [8]. One of the most important is that all *FLT3* internal tandem duplication (ITD)-mutated patients belong to the intermediate-risk category, irrespective of the allelic ratio and a concurrent *NPM1* mutation [9]. *FLT3* mutations are important adverse prognostic factors and key therapeutic targets. However, they are not stable and may arise or disappear at relapse. In addition, certain co-mutation profiles associated with myelodysplasia that confer adverse prognosis to some genetic AML subtypes have been recognized [8].

AML is more complex and heterogeneous than previously thought; therefore, detecting the genetic alterations carried by the different clones causing AML could be very useful for a better understanding of the evolutionary process of the tumor. However, conventional bulk DNA sequencing is insufficient for this purpose, as computational inference using variant allele frequency (VAF) data is limited to accurately deduce the mutation hierarchy and reconstruct clonal structure, and may miss rare or minority populations and individual cell information [10,11]. The best solution to dissect clonal diversity is to obtain genomic information by high-throughput genomic analysis at the single-cell level, as it allows the detection of co-occurrences of mutations in each cell and their order of appearance [10,12]. Prior single-cell DNA sequencing approaches were laborious, with low cell throughput and a high level of artifacts as a result of previous whole-genome amplification [13]. In this study, we used a microfluidic technology that enables the simultaneous characterization of DNA profile and cell-surface proteins in a relapsed *FLT3*-mutated AML patient at two different time-points, at diagnosis and at relapse. This approach has a great potential for defining the genetic alterations that cause AML or confer drug resistance [14,15].

## 2. Case Presentation

A 68-year-old male patient with good general condition and ECOG of 0, was diagnosed in August 2020 with AML M2 subtype according to the French–American–British (FAB) classification [16]. His initial complete blood cell count showed a hemoglobin of 10.6 g/dL, a white blood cell count of 18.8 × 10^9^/L and a platelet count of 75 × 10^9^/L. Bone marrow (BM) showed hypercellularity with 23% small to large blasts, occasionally with “cup-like” nuclear morphology [17,18], and granulocytic hyperplasia with markedly dysgranulopoiesis. Karyotype and fluorescence in situ hybridization were normal. Multiparameter flow cytometry (MFC) was carried out according to EuroFlow standard protocols [19,20], and revealed the presence of two myeloid blast populations expressing immature markers: a predominant population CD34^−/dim^, CD117^dim/+^, HLA-DR^−/+^, CD13^−/++^, CD64^−^, CD33^+/++^, CD38^+^ (P1, 12.5%); and another minor population CD34^++^, CD117^+^, HLA-DR^−/dim^, CD13^dim/+^, CD64^−^, CD33^−/dim^, CD38^−^ (P2, 0.01%) (Figure S1).

Molecular testing was positive for *NPM1* mutation and *FLT3*-ITD with a low allelic ratio (0.07) by fragment analysis, being classified as favorable risk according to the former ELN 2017 criteria [21]. Bulk targeted next-generation sequencing analysis (NGS), performed with a custom Pan-Myeloid Panel (Sophia Genetics, Saint Sulpice, Switzerland) [22] (see Supplementary Materials), revealed a *NPM1* mutation (p.W288Cfs*12) with a VAF of 48%, a subclonal *FLT3*-ITD (p.R595_L601dup) with a VAF of 4.6%, and two co-occurring *DNMT3A* mutations (p.R882H and p.R749G) with a VAF of 44.5% and 47.2%, respectively.

Considering the molecular and clinical findings (Table 1), this patient was included in the Programa Español de Tratamientos en Hematología (PETHEMA) AML registry and was treated according to the LMA2017 protocol, consisting of 3-day idarubicin (12 mg/m^2^) and 7-day cytarabine (200 mg/m^2^) in association with the selective FLT3 inhibitor midostaurin [23]. The patient achieved morphology-based complete remission (CR) with positive minimal residual disease (MRD) by MFC (0.32%), and by reverse transcription-quantitative polymerase chain reaction (RT-qPCR), with 1528 copies of mutant *NPM1* in BM and 827 in peripheral blood (PB). Subsequently, the patient received two cycles of intermediate-dose cytarabine (IDAC) in combination with midostaurin as consolidation chemotherapy, achieving CR with negative MRD after the first IDAC course. An allogeneic hematopoietic stem cell transplantation (allo-HSCT) was planned, but the patient presented a hematologic relapse (6% BM blasts) and consequently, he received salvage therapy (demivistat in combination with high-dose cytarabine (HiDAC) and mitoxantrone) according to the ARMADA 2000 (AML003) clinical trial (NCT03504410).

On day +21 of salvage chemotherapy, hematologic progression (35% BM blasts) was detected, and the patient presented a high copy number of mutated *NPM1*, and a slightly higher ratio (0.10) of the *FLT3*-ITD mutation. NGS analysis showed a small increase in the VAF of the *FLT3*-ITD (12.4%), and the remaining mutations showed a VAF decrease: *NPM1*, 13.2%; *DNMT3A* p.R882H, 25.1%; *DNMT3A* p.R749G, 25.9% (Table 1). At this time point, the patient underwent a sequential conditioning PB allo-HSCT from an HLA-matched sibling donor. After +56 days post-HSCT, even though his achievement of complete donor hematopoietic chimerism, an increase in the number of mutant *NPM1* copies was detected. On day +88 post-HSCT, a PB morphologic relapse (83% blasts) was observed, which was confirmed molecularly, with a significant increase in mutant *NPM1* copies and *FLT3*-ITD ratio (0.84), as well as in the VAF percentage from NGS analysis (*FLT3*-ITD, 46.8%; *NPM1*, 41.7%; *DNMT3A* p.R882H, 45.1%; *DNMT3A* p.R749G, 46.3%) (Table 1). At this moment, the patient was treated with the selective second-generation FLT3 inhibitor gilteritinib, showing a significant blast reduction (3%) after 1 month treatment [24]. One month later, a new hematological progression (44% PB blasts) was observed coinciding with a malabsorption syndrome, caused by an intestinal graft-versus-host-disease (GVHD). One month later, the patient died due to GVHD complications and AML progression.

All procedures in this study were approved by the local Ethical Committee, in accordance with Spanish law and the Declaration of Helsinki. Written informed consent was obtained from the patient. Clinical and biological evolutionary data from the case are described and graphically represented in Table 1 and Figure S2, respectively.

### 2.1. Single-Cell Analysis

**Figure 1:**
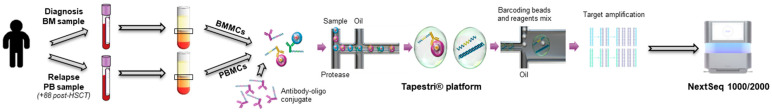
Schematics flowchart of the single-cell DNA sequencing approach using Tapestri platform from Mission Bio, Inc.

To understand the clinical scenario of this patient, simultaneous DNA single-cell sequencing and cell-surface immunophenotyping (scDNAseq and protein-seq) of mononuclear cells (MC) from diagnostic (BMMC) and relapse samples (PBMC) (+88 post-HSCT) was done, using a scDNAseq platform (Tapestri, Mission Bio, Inc. South San Francisco, CA, USA) (Figure 1) [14,15]. To analyze the clonal architecture, scDNAseq was carried through a commercial myeloid gene panel, which included 45 frequently mutated genes (see Supplementary Methods). The phenotypic characterization by protein-seq was performed according to the Mission Bio’s protocol using a custom-designed panel comprising 12 antibodies oligo-conjugated (AOCs) (see Supplementary Methods).

After sequencing and de-multiplexing the diagnostic and relapse samples based on their specific barcodes, sequencing data were processed using the Tapestri Pipeline (Mission Bio, Inc.) and we obtained data from 6941 and 5686 cells, respectively. Data were visualized using the Tapestri Insights software package (version 2.2) and analyzed by an in-house Python code developed by Mission Bio (see Supplementary Methods). After quality filtering, 116 variants from diagnosis and 152 from relapse were retained, considering non-synonymous variants in coding regions, and genotyped in >80% of cells. The same 4 mutations found by bulk targeted NGS in both tumor samples were detected by scDNAseq. The VAFs calculated from bulk NGS and scDNAseq for these specific mutations, were comparable at both time points for this patient (Table 2).

#### 2.1.1. Results at Diagnosis

**Figure 2:**
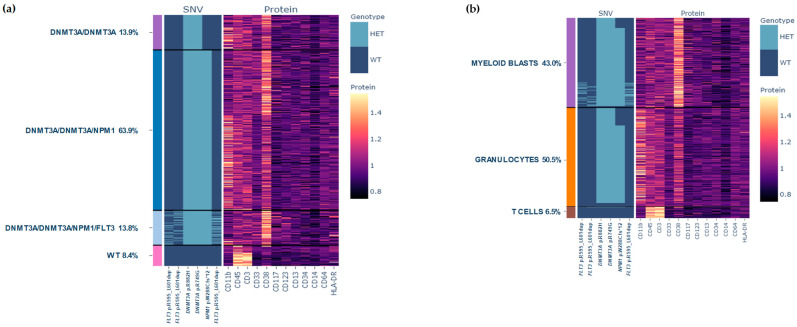
Clonal architecture of AML patient at diagnosis. (**a**) Presence of wild-type (pink) versus *DNMT3A/DNMT3A* (purple), *DNMT3A/DNMT3A/NPM1* (dark blue), and *DNMT3A/DNMT3A/NPM1/FLT3* (light blue) clones in AML patient at diagnosis. (**b**) Different cell populations according to their phenotype: myeloid blasts (purple), granulocytes (orange) and T cells (brown). Rows represent the individual cells and columns represent the regions covered by commercial myeloid gene panel. The amplicons that targeted the *FLT3* gene did not individually cover the ITD (21 bp) of this case, requiring the combination of three amplicons for covering and detecting the complete ITD. Color scale indicates the number of normalized reads for surface protein expression.

Four independent genetically defined clones were identified in the BM: wild-type (WT, 8.4%), *DNMT3A/DNMT3A* (13.9%), *DNMT3A/DNMT3A/NPM1* (63.9%), and *DNMT3A/DNMT3A/NPM1/FLT3* (13.8%) (Figure 2a). Single-cell surface protein characterization identified 20 clusters, which were grouped as immature myeloid blasts (CD38^++^, CD33^+^, CD117^+^ and CD11b^−^), granulocytes (high expression of CD11b), and T cells (CD45^+^, CD3^+^) (Figure 2b).

**Figure 3:**
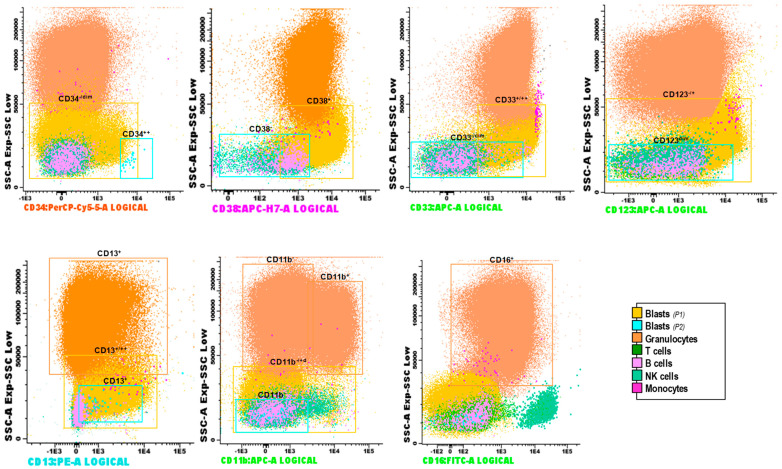
Immunophenotypic features of the different populations identified at the time of diagnosis by multiparameter flow cytometry (MFC), according to antibody custom panel described in Table S4.

Combined genetic and phenotypic data showed that *DNMT3A* and *NPM1* mutations were present not only in myeloid blasts, but also in the granulocytes, which would indicate that they belonged to the neoplastic clone, as there was a certain degree of prior dysplasia in the BM of this patient. To confirm this finding, we sorted the granulocytic population and sequenced it by targeted NGS, demonstrating the presence of the same *DNMT3A* (p.R882H, 47.3%; and p.R749G, 47.0%) and *NPM1* (46.7%) mutations. In contrast, *FLT3*-ITD was subclonal and was found only in the myeloid blast population (Figure 2a,b). The T cell population did not show any mutation, therefore corresponding to the so-called WT clone together with a small proportion of normal granulocytes without mutations (Figure 2b). All these findings were consistent with those of the MFC performed in parallel at the time of diagnosis (Figure 3).

MFC at diagnosis demonstrated the presence of different subsets, including two different types of blasts: a predominant population (P1): 30.4%, CD34^−/dim^, CD38^+^, CD33^+/++^, CD123^−/+^, CD13^+/++^, CD11b^−/dim^; and a minor population (P2): 0.003%, CD34^++^, CD38^−^, CD33^−/dim^, CD123^−/dim^, CD13^+^, CD11b^−^. At the single-cell level, it was not feasible to differentiate both myeloid blasts populations, probably due to the low numbers of the P2 populations and the tiny phenotypic differences, only detectable with highly sensitive MFC. On the other hand, we could detect a large number of granulocytes (70.3%), 63% of them aberrantly lost CD11b as they retain their positivity for CD16 and CD13 (Figure 3), supporting the dysplasia observed in scDNAseq at the genetic level. In addition to myeloid blasts and granulocytes, we identified T cells (2.36%, CD45+ and CD3+) and a small percentage of NK cells, B cells and monocytes, without AOCs to distinguish them by scDNAseq.

#### 2.1.2. Results at Relapse

**Figure 4:**
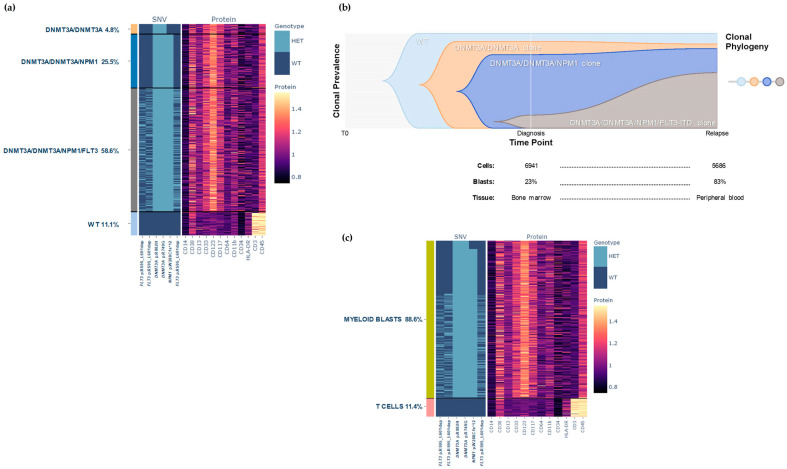
Clonal architecture of AML patient at relapse. (**a**) Presence of wild-type (light blue) versus *DNMT3A/DNMT3A* (yellow), *DNMT3A/DNMT3A/NPM1* (dark blue), and *DNMT3A/DNMT3A/NPM1/FLT3* (dark grey) clones in AML patient at relapse. Rows represent the individual cells and columns represent the regions covered by commercial myeloid gene panel. The amplicons that targeted the *FLT3* gene did not individually cover the ITD (21 bp) of this case, requiring the combination of three amplicons for covering and detecting the complete ITD. Color scale indicates the number of normalized reads for surface protein expression. (**b**) Visualizing hypothesized clonal dynamics by fishplot diagram. The proportions of each clone at each time point were used to predict likely clonal evolution [25]. (**c**) Different cell populations according to their phenotype: myeloid blasts (green) and T cells (pink).

Single-cell analysis of tumor cells at relapse detected the same four clones previously identified. However, at this disease stage the major clone was the quadruple mutant *DNMT3A/DNMT3A/NPM1/FLT3* (58.6%). This clone was the smaller at diagnosis and looked to underwent an expansion upon treatment (Figure 4a,b). In contrast, *DNMT3A/DNMT3A* (4.8%) and *DNMT3A/DNMT3A/NPM1* (25.5%) clones were smaller at relapse (Figure 4b). According to the phenotype, two cell populations were seen: myeloid blasts (88.6%), CD34^−/++^, CD38^+^, CD33^−/++^, CD123^++^, CD117^−/+^, CD11b^−/+^ and CD45^+^, and T-cells (11.4%) typically CD45+ and CD3+ (Figure 4c).

MFC analysis at relapse demonstrated that the main cell population consisted of myeloid blasts (94.2%), which included a combination of the two populations identified at diagnosis (Figure S3), which could not be distinguished by MFC. The number of granulocytes was lower (1.3%) and most of them were mature cells (CD16+). This population showed dysplastic characteristics such us persistence of CD117 positivity (asynchronous maturation) and loss of SSC. In addition, other small populations corresponding to T cells (3.83%), NK cells, B cells and monocytes were identified (Figure S3).

## 3. Discussion

AML is characterized by marked clonal diversity as a result of the accumulation of genetic alterations within tumor resulting in the coexistence of competing clones, highlighting the genetic complexity of the disease [11]. It has been shown that AML progression and relapse may be due to the selection and expansion of resistant clones that escape treatment [26,27].

In this study, we have used a scDNAseq microfluidic approach that allows the simultaneous profiling of DNA and cell-surface proteins, to provide an accurate characterization of the intratumor heterogeneity of AML cells in a relapsed *FLT3*-mutated patient at two different time-points, at diagnosis and at relapse. A 68-year-old male diagnosed with AML and normal karyotype, who was found to have a *FLT3*-ITD with an initial low ratio and a concurrent *NPM1* mutation, therefore categorized as favorable genetic risk according to the ELN 2017 classification, and with no adverse clinical features. He was treated with chemotherapy plus midostaurin and gilteritinib FLT3 inhibitors [23,24], in addition to an allogeneic hematopoietic stem cell transplantation, and presented an unexpected aggressive outcome and final relapse.

**Figure 5:**
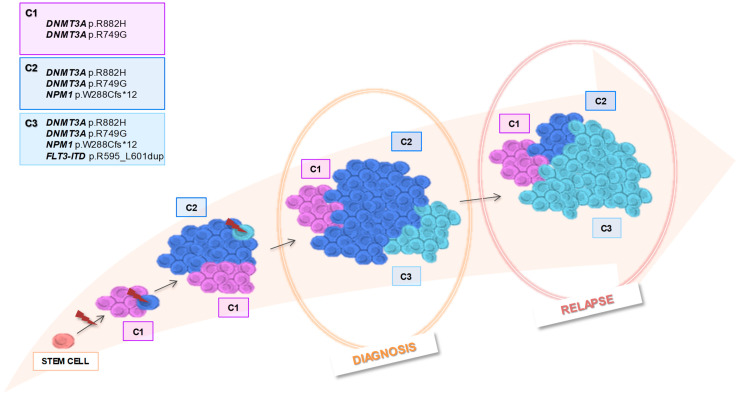
Clonal evolution model of AML patient representing the tumor architecture at different stages of the disease.

We hypothesized that the AML subpopulations present within this patient could be genetically and thereby phenotypically distinct, and these changes could be driving the onset and progression of its disease. Although potential initiating mutations (*FLT3*-ITD, *NPM1* and *DNMT3A*) were present at all time points, the bulk DNA sequencing could not distinguish the different clones associated with treatment resistance or disease progression. The scDNAseq successfully corroborated the number and frequency of variants detected by bulk NGS, and enabled us to determine the co-occurrence pattern and clonal architecture, highlighting a major switch in clonal dominance during the course of the disease, with one of the clones prevailing at diagnosis and a different one at relapse, allowing us to draw a theoretical clonal evolution model (Figure 5). This approach reported interesting data on the order of acquisition of the mutations in our patient: *DNMT3A* mutations (p.R882H and p.R749G) occurred before the *NPM1* mutation (p.W288Cfs*12) and *FLT3*-ITD. Although our report is limited to a single case, these data support the concept that mutations in genes implied in epigenetic regulation are initial events in clonal evolution and they arose from preleukemic progenitor cells, being early founder hits prior to leukemogenic events [3,4]. Furthermore, our data showed that *NPM1* mutation represent a secondary hit in agreement with previous published reports that considered it a leukemogenic event, driving progression to AML [3,4,28]. The presence of both *DNMT3A* and *NPM1* mutations in the granulocytic population indicates their appearance in early stages of hematopoiesis, giving rise to hematopoietic clones that persist over time and survive to different therapies [3,4]. This hypothesis would be supported by the persistence of copies for mutant *NPM1* after allo-HSCT despite successful BM engraftment with a complete donor chimerism, suggesting that the preleukemic clone still exists after allo-HSCT [28,29]. The dysplastic granulocytic immunophenotype observed by MFC supported this genetic data, and could be explained by the presence of high number of these cells after separation by density gradient, because the dysplasia could modify the cell density. In addition to this, the patient’s granulocytic hyperplasia possibly contaminates the mononuclear cell layer making difficult this gradient separation. However, neither bulk NGS nor scDNAseq were able to detect mutations associated with primary resistance to FLT3 inhibitors, such point mutations in genes coding for activating kinases (*FLT3* or *NRAS*/*KRAS*) [10].

The present report shows the *FLT3*-ITD as the late event after *DNMT3A* and *NPM1* mutations, according to other papers that described proliferative mutations in genes involved in signaling as final events in leukemogenesis [28,30]. Therefore, the most likely scenario, confirmed by the fishplot prediction, is that mutations were acquired sequentially in an ancestral clone giving rise to new clonal populations of cells that would coexist with the surviving parental clones. The clone with *FLT3*-ITD (*DNMT3A/DNMT3A/NPM1/FLT3*) was the least represented at diagnosis, it grew progressively until underwent an expansion, being the main responsible clone for the relapse. The absence of treatment with FLT3 inhibitor drugs for a long period of time during the disease course, five months approximately, and the selective pressure of chemotherapy may have contributed to this clonal size increasement, and consequently, to this dismal outcome.

In summary, we show how the Tapestri technology allowed us to identify cell-surface markers that can be used for subclone purification and subsequent scDNAseq [10,12] and demonstrate its usefulness to detect under-represented subclones and to better understand AML resistance mechanisms. We have precisely characterized the clonal architecture of this patient, and have demonstrated the sequence of acquisition of genetic lesions. Detecting minor subpopulations would be a benefit to develop treatment strategies aiming at the eradication of all tumor clones.

## Figures and Tables

**Table 1:** Clinical and biological evolutionary data from the patient.

	Date	Sample Origen	Blasts by Morphology (%)	Blasts by MFC (%)	*FLT3* Ratio	*NPM1* Copies	NGS Analysis (VAF, %)			
							*FLT3* p.R595_L601dup	*NPM1* p.W288Cfs*12	*DNMT3A* p.R882H	*DNMT3* p.R749G
Diagnosis	17 August 2020	BM	23.00	12.50	0.07	157,196.2	4.70	48.00	44.50	47.20
Induction therapy (idarubicin + cytarabine + midostaurin)	19 August 2020									
Day +21	9 September 2020	BM	0	0.01	NA	55,275.9	NA	NA	NA	NA
Day +29	17 September 2020	BM	0	0.21	NA	27,653.7	NA	NA	NA	NA
Consolidation (intermediate-dose cytarabine + midostaurin)	26 September 2020									
Cycle 1	29 October 2020	BM	0	0	NA	1528.1	NA	NA	NA	NA
Cycle 2	26 November2020	BM	0	0.13	Unmutated	4586.1	NA	NA	NA	NA
Relapse	21 Decemebr 2020	BM	6.00	2.70	0.03	108,460.8	NA	NA	NA	NA
Rescue therapy (demivistat + HiDAC + mitoxantrone)	23 Decemebr 2020									
Day +21, progression	13 January 2021	BM	35.00	21.10	0.10	328,495.3	12.40	13.20	25.10	25.90
Allo-HSCT using sequential conditioning										
Pre-HSCT	21 January 2021	BM	Aplasia	Aplasia	NA	63,591.9	NA	NA	NA	NA
Day +24 post-HSCT	23 February 2021	BM	0	0	Unmutated	19.3	NA	NA	NA	NA
Day +56 post-HSCT	29 March 2021	BM	0	0	Unmutated	9555.6	NA	NA	NA	NA
Day +88 post-HSCT, relapse	27 April 2021	PB	83.00	-	0.84	335,980.0	46.80	41.70	45.10	46.30
Rescue therapy (gilteritinib)	12 May 2021									
Day +30	11 June 2021	PB	3.00	-	-	-	-	-	-	-
Day +61, progression	13 July 2021	PB	43.60	-	0.89	275,722.5	NA	NA	NA	NA
Day +74	26 July 2021	PB	10.00	-	-	-	-	-	-	-
Day +77	29 July 2021	PB	13.90	-	-	-	-	-	-	-
Day +80	1 August 2021	PB	16.70	-	-	-	-	-	-	-
Death	20 August 2021	PB	NA	-	0.87	480,703.3	NA	NA	NA	NA

Allo-HSCT, allogeneic hematopoietic stem-cell transplant; BM, bone marrow; MFC, multiparameter flow cytometry; NA, not assessed; HiDAC, histone deacetylase inhibitor; NGS, next-generation sequencing; PB, peripheral blood; VAF, variant allelic frequency. The dashes mean no sample available.

**Table 2:** Comparison of variant allele frequency (VAF) by both techniques, bulk NGS and scDNAseq.

Mutations	Diagnosis		Relapse	
	Bulk NGS (%)	scDNAseq (%)	Bulk NGS (%)	scDNAseq (%)
*DNMT3A* p.R882H	44.5	48.3	45.1	46.8
*DNMT3A* p.R749G	47.2	46.9	46.3	46.3
*NPM1* p.W288Cfs*12	48.0	42.9	41.7	43.7
*FLT3* p.R595_L601dup	4.6	7.4	46.8	50.5

## Data Availability

No new data were created or analyzed in this study.

## Supplementary Materials

The following supporting information can be downloaded at: https://www.mdpi.com/article/10.3390/biomedicines12010066/s1: Materials and Methods, Supplementary Tables and Supplementary Figures; Figure S1: Immunophenotypic features of the different populations identified at the time of diagnosis by multiparameter flow cytometry in bone marrow sample, according to EuroFlow standard protocols; Figure S2: Progression of the percentage of blasts, number of NPM1 copies and FLT3-ITD allelic ratio in time; Figure S3: Immunophenotypic features of the different populations identified at the time of relapse by multiparameter flow cytometry, according to antibody custom panel described in Table S4. Table S1: Pan-Myeloid Panel target regions per gene; Table S2: Mision Bio’s Tapestri 45-gene myeloid panel; Table S3: Custom AOC panel; Table S4: Antibody panel employed for the evaluation of the BMMCs and PBMCs.

## References

[1] Döhner H., Weisdorf D.J., Bloomfield C.D. (2015). Acute Myeloid Leukemia. N. Engl. J. Med..

[2] Ley T.J., Miller C., Ding L., Raphael B.J., Mungall A.J., Robertson A., Hoadley K., Triche T.J., Laird P.W., Cancer Genome Atlas Research Network (2013). Genomic and epigenomic landscapes of adult de novo acute myeloid leukemia. N. Engl. J. Med..

[3] Shlush L.I., Zandi S., Mitchell A., Chen W.C., Brandwein J.M., Gupta V., Kennedy J.A., Schimmer A.D., Schuh A.C., Yee K.W. (2014). Identification of pre-leukaemic haematopoietic stem cells in acute leukaemia. Nature.

[4] Bullinger L., Döhner K., Döhner H. (2017). Genomics of Acute Myeloid Leukemia Diagnosis and Pathways. J. Clin. Oncol..

[5] Welch J.S., Ley T.J., Link D.C., Miller C.A., Larson D.E., Koboldt D.C., Wartman L.D., Lamprecht T.L., Liu F., Xia J. (2012). The origin and evolution of mutations in acute myeloid leukemia. Cell.

[6] Khoury J.D., Solary E., Abla O., Akkari Y., Alaggio R., Apperley J.F., Bejar R., Berti E., Busque L., Chan J.K.C. (2022). The 5th edition of the World Health Organization Classification of Haematolymphoid Tumours: Myeloid and Histiocytic/Dendritic Neoplasms. Leukemia.

[7] Arber D.A., Orazi A., Hasserjian R.P., Borowitz M.J., Calvo K.R., Kvasnicka H.-M., Wang S.A., Bagg A., Barbui T., Branford S. (2022). International Consensus Classification of Myeloid Neoplasms and Acute Leukemias: Integrating morphologic, clinical, and genomic data. Blood.

[8] Döhner H., Wei A.H., Appelbaum F.R., Craddock C., DiNardo C.D., Dombret H., Ebert B.L., Fenaux P., Godley L.A., Hasserjian R.P. (2022). Diagnosis and management of AML in adults: 2022 recommendations from an international expert panel on behalf of the ELN. Blood.

[9] Döhner K., Thiede C., Jahn N., Panina E., Gambietz A., Larson R.A., Prior T.W., Marcucci G., Jones D., Krauter J. (2020). Impact of NPM1/FLT3-ITD genotypes defined by the 2017 European LeukemiaNet in patients with acute myeloid leukemia. Blood.

[10] McMahon C.M., Ferng T., Canaani J., Wang E.S., Morrissette J.J., Eastburn D.J., Pellegrino M., Durruthy-Durruthy R., Watt C.D., Asthana S. (2019). Clonal Selection with RAS Pathway Activation Mediates Secondary Clinical Resistance to Selective FLT3 Inhibition in Acute Myeloid Leukemia. Cancer Discov..

[11] Romer-Seibert J.S., Meyer S.E. (2021). Genetic heterogeneity and clonal evolution in acute myeloid leukemia. Curr. Opin. Hematol..

[12] Morita K., Wang F., Jahn K., Hu T., Tanaka T., Sasaki Y., Kuipers J., Loghavi S., Wang S.A., Yan Y. (2020). Clonal evolution of acute myeloid leukemia revealed by high-throughput single-cell genomics. Nat. Commun..

[13] Wang Y., Navin N.E. (2015). Advances and applications of single-cell sequencing technologies. Mol. Cell.

[14] Pellegrino M., Sciambi A., Treusch S., Durruthy-Durruthy R., Gokhale K., Jacob J., Chen T.X., Geis J.A., Oldham W., Matthews J. (2018). High-throughput single-cell DNA sequencing of acute myeloid leukemia tumors with droplet microfluidics. Genome Res..

[15] Demaree B., Delley C.L., Vasudevan H.N., Peretz C.A.C., Ruff D., Smith C.C., Abate A.R. (2021). Joint profiling of DNA and proteins in single cells to dissect genotype-phenotype associations in leukemia. Nat. Commun..

[16] Bennett J.M., Catovsky D., Daniel M.T., Flandrin G., Galton D.A.G., Gralnick H.R., Sultan C. (1985). Proposed revised criteria for the classification of acute myeloid leukemia. A report of the French-American-British Cooperative Group. Ann. Intern. Med..

[17] Kussick S.J., Stirewalt D.L., Yi H.S., Sheets K.M., Pogosova-Agadjanyan E., Braswell S., Norwood T.H., Radich J.P., Wood B.L. (2004). A distinctive nuclear morphology in acute myeloid leukemia is strongly associated with loss of HLA-DR expression and *FLT3* internal tandem duplication. Leukemia.

[18] Park B.G., Chi H.-S., Jang S., Park C.-J., Kim D.-Y., Lee J.-H., Lee J.-H., Lee K.-H. (2013). Association of cup-like nuclei in blasts with *FLT3* and *NPM1* mutations in acute myeloid leukemia. Ann. Hematol..

[19] van Dongen J.J.M., Lhermitte L., Böttcher S., Almeida J., van der Velden V.H.J., Flores-Montero J., Rawstron A., Asnafi V., Lécrevisse Q., Lucio P. (2012). EuroFlow antibody panels for standardized n-dimensional flow cytometric immunophenotyping of normal, reactive and malignant leukocytes. Leukemia.

[20] Kalina T., Flores-Montero J., van der Velden V.H.J., Martin-Ayuso M., Böttcher S., Ritgen M., Almeida J., Lhermitte L., Asnafi V., Mendonça A. (2012). EuroFlow standardization of flow cytometer instrument settings and immunophenotyping protocols. Leukemia.

[21] Döhner H., Estey E., Grimwade D., Amadori S., Appelbaum F.R., Büchner T., Dombret H., Ebert B.L., Fenaux P., Larson R.A. (2017). Diagnosis and management of AML in adults: 2017 ELN recommendations from an international expert panel. Blood.

[22] Aguilera-Diaz A., Vazquez I., Ariceta B., Mañú A., Blasco-Iturri Z., Palomino-Echeverría S., Larrayoz M.J., García-Sanz R., Prieto-Conde M.I., Chillón M.d.C. (2020). Assessment of the clinical utility of four NGS panels in myeloid malignancies. Suggestions for NGS panel choice or design. PLoS ONE.

[23] Stone R.M., Mandrekar S.J., Sanford B.L., Laumann K., Geyer S., Bloomfield C.D., Thiede C., Prior T.W., Döhner K., Marcucci G. (2017). Midostaurin plus chemotherapy for acute myeloid leukemia with a FLT3 mutation. N. Engl. J. Med..

[24] Perl A.E., Martinelli G., Cortes J.E., Neubauer A., Berman E., Paolini S., Montesinos P., Baer M.R., Larson R.A., Ustun C. (2019). Gilteritinib or Chemotherapy for Relapsed or Refractory *FLT3*-Mutated AML. N. Engl. J. Med..

[25] Smith M.A., Nielsen C.B., Chan F.C., McPherson A., Roth A., Farahani H., Machev D., Steif A., Shah S.P. (2017). E-scape: Interactive visualization of single-cell phylogenetics and cancer evolution. Nat. Methods.

[26] Greaves M., Maley C.C. (2012). Clonal evolution in cancer. Nature.

[27] Walter M.J., Shen D., Ding L., Shao J., Koboldt D.C., Chen K., Larson D.E., McLellan M.D., Dooling D., Abbott R. (2012). Clonal architecture of secondary acute myeloid leukemia. N. Engl. J. Med..

[28] Saeed B.R., Manta L., Raffel S., Pyl P.T., Buss E.C., Wang W., Eckstein V., Jauch A., Trumpp A., Huber W. (2021). Analysis of nonleukemic cellular subcompartments reconstructs clonal evolution of acute myeloid leukemia and identifies therapy-resistant preleukemic clones. Int. J. Cancer.

[29] Xu L., Durruthy-Durruthy R., Eastburn D.J., Pellegrino M., Shah O., Meyer E., Zehnder J. (2019). Clonal Evolution and Changes in Two AML Patients Detected with A Novel Single-Cell DNA Sequencing Platform. Sci. Rep..

[30] Corces-Zimmerman M.R., Hong W.-J., Weissman I.L., Medeiros B.C., Majeti R. (2014). Preleukemic mutations in human acute myeloid leukemia affect epigenetic regulators and persist in remission. Proc. Natl. Acad. Sci. USA.

