# Supplementary material for: Single-Cell DNA Sequencing and Immunophenotypic Profiling to Track Clonal Evolution in an Acute Myeloid Leukemia Patient
Source: Biomedicines. 2023 Dec 27;12(1):66. doi: 10.3390/biomedicines12010066 (PMC10813288; doi:10.3390/biomedicines12010066)
Supplement: Supplementary file 1 [file biomedicines-12-00066-s001.zip › biomedicines-2772741-supplementary.pdf]

SUPPLEMENTARY MATERIALS

Case Report

Single-Cell DNA Sequencing and Immunophenotypic Profiling to Track Clonal Evolution in an Acute Myeloid Leukemia Patient.

María García-Álvarez [1], Ana Yeguas [2], Cristina Jiménez [1], Alejandro Medina-Herrera [1], Verónica González-Calle [1], Montserrat Hernández-Ruano [1], Rebeca Maldonado [1], Irene Aires [1], Cristina Casquero [1], Inmaculada Sánchez-Villares [1], Ana Balanzategui [1], María Eugenia Sarasquete [1], Miguel Alcoceba [1], María Belén Vidriales [1], Marcos González-Díaz [1], Ramón García-Sanz [1,*] and María Carmen Chillón [1]

[1] Hematology Department, University Hospital of Salamanca (HUS/IBSAL), CIBERONC and Cancer Research Institute of Salamanca-IBMCC (USAL-CSIC), Salamanca, 37007, Spain
[2] Hematology Department, Complejo Asistencial Universitario de Palencia, Palencia, 34005, Spain
* Correspondence: rgarcias@usal.es

CONTENTS:

1. **Materials and Methods**
2. **Supplementary Tables**
3. **Supplementary Figures**
4. **References**

1. Material and methods

Clinical samples

Bone marrow (BM) aspirate and peripheral blood (PB) samples were collected on EDTA anticoagulant from the patient at diagnosis and relapse, respectively, after receipt of written informed consent approved by the local Ethical Committee, in accordance with Spanish law and the Declaration of Helsinki. Viable mononuclear cells from fresh BM (BMMCs) and PB (PBMCs) samples were purified using a separation by density gradient and frozen them on fetal bovine serum (FBS) containing 10% dimethyl sulfoxide (DMSO) at -80 ºC.

Genomic DNA (gDNA) from fresh BM and PB samples were isolated by the automated DNA extractor Maxwell® 16 System (Promega, Madison, WI, USA).

Bulk targeted next-generation sequencing (NGS)

Bulk targeted sequencing was performed with a custom Pan-Myeloid Panel (PMP), (SOPHiA GENETICS SA, Saint Sulpice, Switzerland), that covers 59 genes frequently mutated in myeloid pathology (Supplementary Table S1), starting from 200 ng gDNA.[1] Final libraries quantity was measured in a Qubit® 3.0 Fluorometer (Invitrogen™, Eugene, OR, USA), using the dsDNA HS (High Sensitivity) Assay kit, and libraries quality was assessed with a 4200 TapeStation (Agilent Technologies, Santa Clara, CA, USA) system, using the High Sensitivity D1000 assay. Libraries were then normalized, pooled, and 2x301 bp paired-end sequenced on a MiSeq platform (Illumina Inc., San Diego, CA, USA). Raw sequencing FASTQ files were analysed using SOPHiA DDM™ Platform, taking into account somatic single-nucleotide variants (SNVs) and small insertions/deletions (indels) with >300X reads and variant allele frequency ≥3%. Variants in the non-coding region, synonymous variants and single nucleotide polymorphisms (MAF>1%) were excluded.

Single-Cell DNA and Protein Sequencing

Single-cell DNA and protein sequencing was performed using a novel tow-step microfluidic droplet workflow by Mission Bio Tapestri single-cell sequencing platform (Mission Bio, Inc., South San Francisco, CA, USA), according to the manufacturer's instructions.[2] We employed the Mission Bio's Tapestri Myeloid panel, which covering hotspot mutations in 45 genes (312 amplicons) commonly mutated in myeloid malignancies (Supplementary Table S2) for single-cell DNA sequencing. For protein sequencing, antibody-oligo conjugates (AOC) custom panel for targeting cell-surface proteins of interest were designed by us and manufactured by Mission Bio, Inc (Supplementary Table S3). Close to 825,000 thawed BMMCs and PBMCs were incubated with AOC pool for staining (30 min at room temperature), followed by three washes with Dulbecco's phosphate-buffered saline (DPBS) containing 5% FBS. Approximately 125,000 stained cells were resuspended in cell buffer for encapsulation, lysis, protein digest and cell barcoding on the Tapestri platform. Then, targeted DNA region and antibody-oligo tag were amplified by incubating barcoded DNA emulsion in a thermocycler. Emulsions were broken and PCR product was cleaned by enzymatic digest. DNA was purified with 0.7X AMPure XP reagent (Beckman Coulter, Pasadena, CA, USA), which allowed us to separate DNA targets bound to pelleted beads from the antibody DNA tags retained in the supernatant. The supernatant was incubated

with a biotinylated capture oligo at 96ºC for 5 min, transferred immediately to ice for 5 min and captured with streptavidin beads (Dynabead MyOne Streptavidin C1, Thermo Fisher). Then, we amplified DNA libraries with Mission Bio V2 Index Primers and protein libraries bound to streptavidin beads with i5 and i7 index primers in the thermocycler. Finally, libraries were purified with 0.69X and 0.9X AMPure XP reagent (Beckman Coulter), respectively, and their quality was assessed with a 4200 TapeStation (Agilent Technologies) system, using the High Sensitivity D1000 assay. Libraries were quantified with Qubit® 3.0 Fluorometer, pooled and 2x150-bp paired-end sequenced on a NextSeq 1000 (Illumina, Inc.). A 10% ratio of PhiX DNA was used in the sequencing runs. FASTQ files were processed by Tapestri pipeline (Mission Bio, Inc.) and the resulting .loom and .h5 files were visualized using the Tapestri Insights software package and analysed by specific python scripts. After quality filtering, we considered non-synonymous variants in coding regions and genotyped in >80% of cells. The amplicons that targeted the *FLT3* gene could not be individually covered the ITD (21 bp) of this case, requiring the combination of three amplicons for covering and detecting the complete ITD.

Multiparameter flow cytometry (MFC)

BM aspirate from diagnosis was collected on EDTA and 200 µl were stained using the acute leukaemia orientation tube (ALOT) and the seven EuroFlow (EF) acute myeloid leukaemia (AML)/myelodysplastic syndrome (MDS) antibody panel.[3] Sample processing and sample acquisition were carried out according to EF standard protocol.[4] Stained cells were measured in FACSCanto II flow cytometer (BD Bioscience, San Jose, CA, USA), equipped with FACSDiva™ software (BD Bioscience). Data were analysing by Infinicyt v2.0 software (Cytognos, Salamanca, Spain). Thawed BMMCs and PBMCs, from diagnosis and relapse respectively, were resuspended in 200 µl of phosphate-buffered saline (PBS) and stained with a specific antibody panel (Supplementary Table S4). This antibody panel was designed based on diagnosis BM phenotype and antibodies included in AOC custom panel for single-cell analysis. After staining, cell pellet was washed with PBS, centrifuged (5 min at 2000 rpm) and finally resuspended in 200 µl of PBS, and measured in FACSCanto II flow cytometer (BD Bioscience) at a medium flow rate mode. Data were analysing by Infinicyt v2.0 software (Cytognos).

2. Supplementary tables

Table S1. Pan-Myeloid Panel (PMP) target regions per gene. PMP panel design includes a total of 59 genes for SNV and indels.

GENE	Cytoband	NM_	EXONS Target Region
ACD	16q22.1	NM_022914	CDS
ANKRD26	10p12.1	NM_014915	5'UTR + exons 1-2
ASXL1	20q11.21	NM_015338.5	exon 13
ATG2B	14q32.2	NM_018036	exons 28,29,30,31,32,33
ATRX	Xq21.1	NM_000489.4	exons 8-10, 17-31
BCOR	Xp11.4	NM_001123385	CDS
BCORL1	Xq26.1	NM_021946	CDS
CALR	19p13.13	NM_004343.3	exon 9
CBL	11q23.3-qter	NM_005188.3	exons 8-9
CEBPA	19q13.1	NM_004364.4	CDS
CSF3R	1p35-p34.3	NM_156039	exons 14-17
CSNK1A1	5q32	NM_001025105	CDS
CUX1	7q22.1	NM_001202543	CDS
DDX41	5q35.3	NM_016222	CDS
DHX34	19q13.32	NM_014681	exons 2-17
DNMT3A	2p23.3	NM_175629.2	CDS
ETNK1	12p12.1	NM_018638.4	exon 3
ETV6	12p13.2	NM_001987.4	CDS
EZH2	7q35-q36	NM_004456.4	CDS
FLT3	13q12.2	NM_004119.2	exons 11-20
GATA1	Xp11.23	NM_002049.3	exon 2
GATA2	3q21.3	NM_032638.4	CDS
GSKIP	14q32.2	NM_016472	CDS
IDH1	2q32-qter	NM_001282387	exon 4
IDH2	15q21-qter	NM_002168.3	exon 4
IKZF1	7p12.2	NM_006060.5	CDS
JAK2	9p24.1	NM_004972.3	exons 12-15
KIT	4q11-q12	NM_000222.2	exons 2, 8-11, 13, 14, 17, 18
KMT2A	11q23.3	NM_001197104.1	CDS
KRAS	12p12.1	NM_033360	exons 2-4
MBD4	3q21.3	NM_003925	CDS
MECOM	3q26.2	NM_004991	CDS
MPL	1p34.2	NM_005373.2	exons 3-6,10,12
NF1	17q11.2	NM_001042492	CDS
NPM1	5q35.1	NM_002520.6	exons 10, 11
NRAS	1p13.2	NM_002524.4	exons 2-4
PHF6	Xq26.2	NM_032458	CDS
PPM1D	17q23.2	NM_003620	CDS
PTPN11	12q24.13	NM_002834.3	exons 3, 7, 13
RAD21	8q24.11	NM_006265.2	CDS
RUNX1	21q22.3	NM_001754.4	CDS
SAMD9	7q21.2	NM_017654	CDS
SAMD9L	7q21.2	NM_152703	CDS
SETBP1	18q21.1	NM_015559.2	aa850-928 of exon 4
SF3B1	2q33.1	NM_012433.2	exons 11-16
SH2B3/LNK	12q24.12	NM_005475	CDS
SMC1A	Xp11.22	NM_006306	exons 2,11,16,17
SMC3	10q25.2	NM_005445	exons 10, 13, 19, 23, 25, 28
SRP72	4q12	NM_006947	CDS

SRSF2	17q25.1	NM_003016.4	CDS
STAG2	Xq25	NM_001042749.2	CDS
TCL1A	14q32.2	NM_021966	CDS
TERC	3q26.2	NR_001566	Full
TERT	5p15.33	NM_198253	CDS
TET2	4q24	NM_001127208	CDS
TP53	17p13.1	NM_000546.5	CDS
U2AF1	21q22.3	NM_006758	exons 2, 6
WT1	11p13	NM_024426.4	exons 7, 9
ZRSR2	Xp22.1	NM_005089.3	CDS

Table S2. Mision Bio's Tapestri 45-gene myeloid panel

ASXL1	*ERG*	*KDM6A*	*NRAS*	*SMC1A*
ATM	*ETV6*	*KIT*	*PHF6*	*SMC3*
BCOR	*EZH2*	*KMT2A*	*PPM1D*	*STAG2*
BRAF	*FLT3*	*KRAS*	*PTEN*	*STAT3*
CALR	*GATA2*	*MPL*	*PTPN11*	*TET2*
CBL	*GNAS*	*MYC*	*RAD21*	*TP53*
CHEK2	*IDH1*	*MYD88*	*RUNX1*	*U2AF1*
CSF3R	*IDH2*	*NF1*	*SETBP1*	*WT1*
DNMT3A	*JAK2*	*NPM1*	*SF3B1*	*ZRSR2*

Table S3. Custom AOC panel

CD11b	CD117	CD33	CD45
CD13	CD123	CD34	CD64
CD14	CD3	CD38	HLA-DR

Table S4. Antibody panel employed for the evaluation of the BMMCs and PBMCs. The different colours represent the fluorescence colour emitted by each fluorochrome.

**Tube**	**FITC**	**PE**	**PerCPCy5.5**	**PECy7**	**APC**	**APC-H7**	**V450**	**OC515**
**1**	**CD15**<br>Clone MMA<br>BD Bioscience<br>(5 µl)	**CD13**<br>Clone L138<br>BD Bioscience<br>(5 µl)	**CD34**<br>Clone 8G12<br>BD Bioscience<br>(5 µl)	**CD117**<br>Clone 104D2D1<br>Beckman Coulter<br>(5 µl)	**CD33**<br>Clone P67.6<br>BD Bioscience<br>(10 µl)	**CD38**<br>Clone HB7<br>BD Bioscience<br>(3 µl)	**HLA-DR**<br>Clone L243<br>BD Bioscience<br>(5 µl)	**CD45**<br>Clone HI30<br>Immunostep<br>(5 µl)
**2**	**CD15**<br>Clone MMA<br>BD Bioscience<br>(5 µl)	**CD64**<br>Clone 22<br>Cytognos<br>(10 µl)	**CD34**<br>Clone 8G12<br>BD Bioscience<br>(5 µl)	**CD117**<br>Clone 104D2D1<br>Beckman Coulter<br>(5 µl)	**CD11b**<br>Clone D12<br>BD Bioscience<br>(5 µl)	**CD14**<br>Clone MøP9<br>BD Bioscience<br>(5 µl)	**HLA-DR**<br>Clone L243<br>BD Bioscience<br>(5 µl)	**CD45**<br>Clone HI30<br>Immunostep<br>(5 µl)
**3**	**CD16**<br>Clone 3G8<br>Beckman Coulter<br>(20 µl)	**CD13**<br>Clone L138<br>BD Bioscience<br>(5 µl)	**CD34**<br>Clone 8G12<br>BD Bioscience<br>(5 µl)	**CD117**<br>Clone 104D2D1<br>Beckman Coulter<br>(5 µl)	**CD123**<br>Clone AC145<br>Miltenyi Biotec<br>(2 µl)	**CD3**<br>Clone SK7<br>BD Bioscience<br>(5 µl)	**HLA-DR**<br>Clone L243<br>BD Bioscience<br>(5 µl)	**CD45**<br>Clone HI30<br>Immunostep<br>(5 µl)

3. Supplementary figures

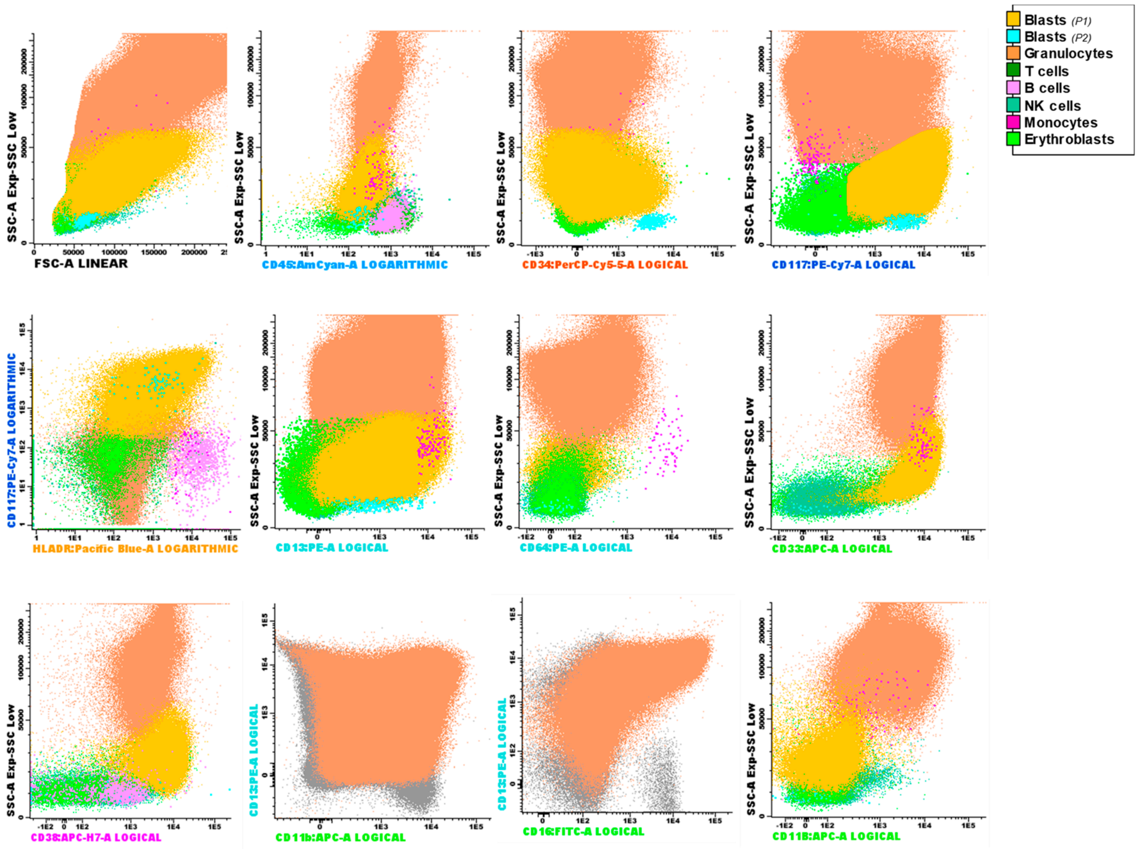

Figure S1. Immunophenotypic features of the different populations identified at the time of diagnosis by multiparameter flow cytometry in bone marrow sample, according to EuroFlow standard protocols.

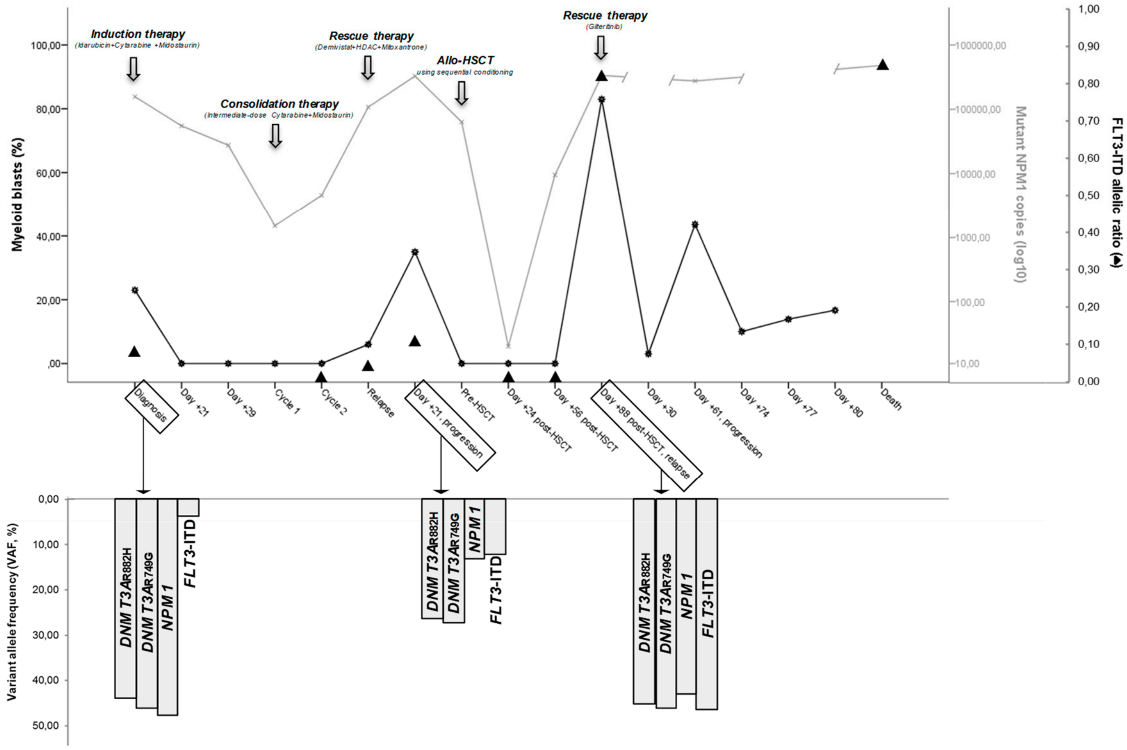

Figure S2. Progression of the percentage of blasts (black line), number of *NPM1* copies (grey line) and *FLT3*-ITD allelic ratio (black triangle) in time. At the bottom of the graph is represented the variant allele frequency of different mutations detected by NGS in three time points.

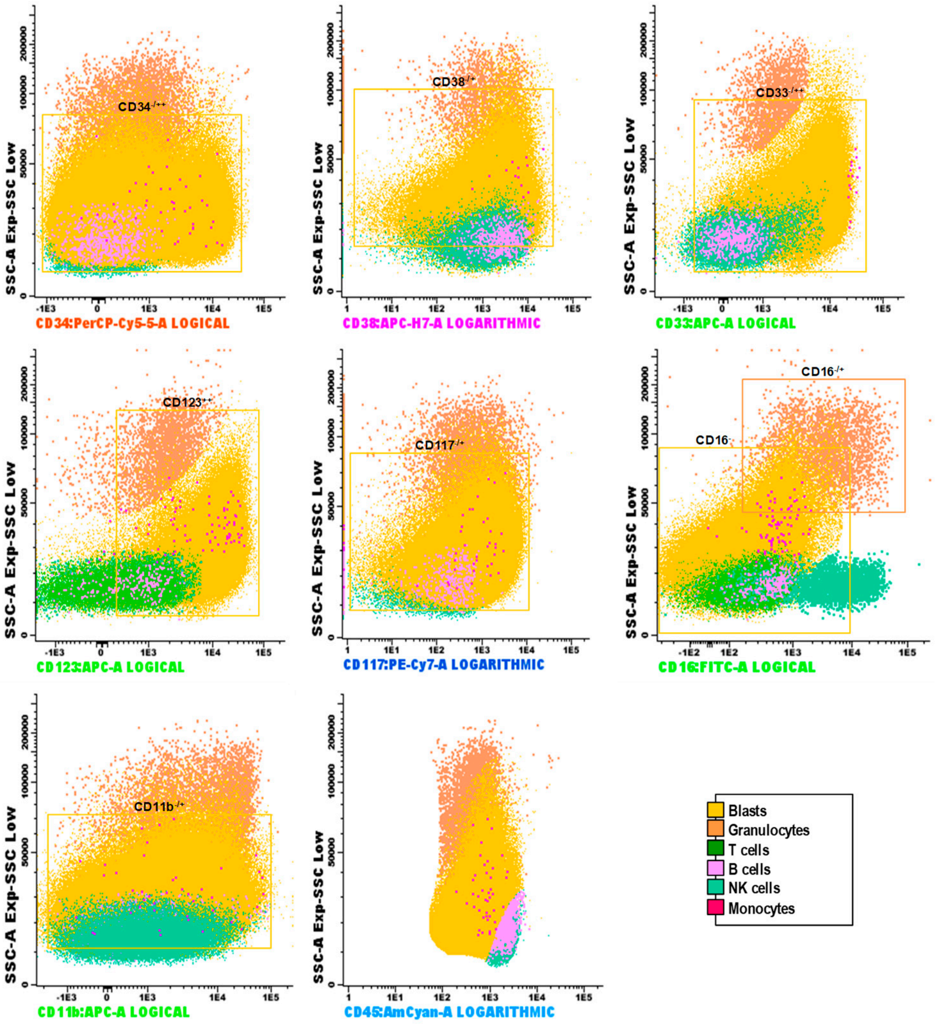

Figure S3. Immunophenotypic features of the different populations identified at the time of relapse by multiparameter flow cytometry in peripheral blood sample using antibody custom panel described in Table S4.

4. References

1. Aguilera-Diaz A, Vazquez I, Ariceta B, et al. Assessment of the clinical utility of four NGS panels in myeloid malignancies. Suggestions for NGS panel choice or design. PLoS One 2020;15(1):e0227986.

2. Pellegrino M, Sciambi A, Treusch S et al. High-throughput single-cell DNA sequencing of acute myeloid leukemia tumors with droplet microfluidics. Genome Res 2018;28:1345-1352.

3. van Dongen JJ, Lhermitte L, Böttcher S et al. EuroFlow antibody panels for standardized n-dimensional flow cytometric immunophenotyping of normal, reactive and malignant leukocytes. Leukemia 2012;26:1908-1975.

4. Kalina T, Flores-Montero J, van dV, V et al. EuroFlow standardization of flow cytometer instrument settings and immunophenotyping protocols. Leukemia 2012;26:1986-2010.
